# The Effect of Loneliness on Social Networking Sites Use and Its Related Behaviors

**DOI:** 10.5539/gjhs.v8n8p162

**Published:** 2015-12-17

**Authors:** Samira Ranaeiy, Mohammad Reza Taghavi, Mohammad Ali Goodarzi

**Affiliations:** 1 Department of Clinical Psychology, Shiraz University, Shiraz, Iran

**Keywords:** socialnetworking sites, loneliness, internet behaviors, reasons of S.N.S use

## Abstract

**Introduction:**

The current research was conducted to examine the effect of “Loneliness”, on time spent in Social Networking Sites (S.N.S), main reasons for S.N.S use, and its related behaviors.

**Materials and Methods:**

156 students of Shiraz University voluntarily participated in this research. Loneliness was assessed usingthe UCLA Loneliness scale. 25% of highest scoring students reported that they were lonely whereas 25% of the lowest scoring students were considered to be non-lonely. The positive and negative reasons of using S.N.S were assessed based on Reasons for Internet Use Scale, and internet behaviors were assessed based on Scale of Internet Behaviors.

**Results:**

There was no difference in time spent in S.N.S as well as the positive and negative reasons of using S.N.S (contrary to literature), but internet behaviors showed a significant difference between “lonely” and “non-lonely” individuals. “Lonely” and “non-lonely” individuals showed a significant difference in “social aspect” of S.N.S behaviors. There was also a significant difference between “Lonely” and “non-Lonely” individuals in “Negative impact” of S.N.S behaviors. Yet, there seemed to be no difference in “competency and convenience aspect” of S.N.S behaviors.

**Conclusions:**

This study suggested that there is no difference between lonely and non-lonely individuals in reasons for using S.N.S and time spent in S.N.S. This finding stands contrary to previous research findings and general literature on the subject In other words, what drives people to S.N.S at the first place shows no significant difference between lonely and non-lonely individuals while after attending S.N.S, social behavior of lonely individuals shows a significant difference which is consistently enhanced online. Lonely people also significantly develop internet-related problems in their daily functioning, including interference with real life socializing.

## 1. Introduction

Using Social Networking Sites (S.N.S) could be considered as one of the most common activities of today’s world. Although S.N.S is widely used to communicate with others, loneliness has long been associated with the use of S.N.S. Early studies indicated that Lonely people turned first to internet and then to S.N.S (Shotten, 1991; Turkle, 1984). The research conducted in the following years focused on the following question: what, if any pathological effects do preference for online communications have on offline social relationships, and what outcomes may result (Joison, 2004; Thayer & Ray, 2006). In 2008, Facebook reported having 67 million active users (those who returned to the site within the last 30 days), with more than half of them returning daily and spending average of 20 minutes per day on the sites (Facebook, 2008). Since S.N.S (e.g. Facebook) has allowed college students population to form friends on the site, it has been successful in providing them pathway to bridge their online and offline contacts (Comscore, 2008). Much of the existing academic research on S.N.S focused on privacy concern and identity presentation, looking at the amount of information and the lack of privacy control enhanced due to problematic Internet Use (PIU) (Grass & Acquisti, 2005). It is argued that user maybe putting themselves at risk both online (e.g. stalking) and offline (e.g. Identity theft).

The present study attempted to uncover an underlying process to explain how psychological variables, specifically loneliness, might affect the reasons of using S.N.S and its related behaviors. In this study we explored the differences between lonely and non-lonely individuals in what drives them first to use S.N.S and then compare their internet behaviors. It seems that for adolescents whose real-life may be troubled by unsatisfactory personal relations or lack of social support, S.N.S may provide a safe and secure interaction. This so-called freedom to recreate or to obscure some aspects of the self-online allows the exploration and expression of multiple and fragmented selves of human existence. S.N.S provides people with fluid identity, anonymity disinhibition and invisibility. This study attended to find predictors of preference of S.N.S, loneliness; the loneliness which is experienced by some of psychological distress. In broad terms, loneliness is defined as a sense of deprivation in one’s social relationships (Murphy & Kupshik, 1992).

## 2. Literature Review, Hypothesis and Research Question

Prior research showed that age, education and gender are related to internet use and network size, social support, and loneliness. People with higher education often have more access to the internet, partlydue toeducational facilities. Women differ from men with respect to internet use (Boneva, Kraut, & Frohlich, 2001). Lonely individuals have reported feeling less ‘competent’ psychologically) (Leung, 2001; Spitzbery & Canary, 1985). Monahan-Martin and Schumacher (2003) found that lonely people are more likely to use internet and email for emotional support than others. Many turned to the internet to escape from the so-called pressures and discomfort of their lives. As internet use spread to a broader population, early Chroniclers of the life online, such as Reingold (1993) and Turkle (1984), continued to draw anecdotal links between loneliness and internet use and abuse. Quantitativestudies that followed confirmed the association betweenloneliness and increased internet use (Kraut, Patterson, Landmark, Kiesler, Mukophadhyay, & Scherlis, 1998; Lavin, Mavtin, Mclarneg, Nola, & Scott. 1999) and compulsive use of the internet (Loytskert & Aiello 1997; Morahan-Martin & Schumacher, 2000; Young, 1998).

A review of some surveys which were conducted in the following years on social communication by the Internet indicates thathigher educated individuals and young adults more easily adopt new communication technologies than others (De Haan & Huysmans, 2006). It is also documented that virtual interactions are very weak compare withface to face interaction, and in the long run it makes the over users to become lonelier (Shojaee et al., 2008).

Kim, La Rose, and Peng (2009) showed that instead of relieving their original problems, individual who were lonely could develop strong compulsive internet use behaviors resulting in negative life outcomes. Blink & Simahel (2009) showed that internet allows adolescents to grasp their identity more easily and intensively, thus the freedom to experiment with self-expression is attractive to them.

### 2.1 Focus of the Current Study

This study wasa comparison of lonely and non-lonely individualsin terms oftime spent onS.N.S, their reasonsforS.N.S use, and the way S.N.S affects their interactions. It is hypothesized that compared to non-lonely individuals, lonely people would be more likely to spend time onS.N.S, the reasons (positive and negative) for S.N.S use are to be significantly different, and they would show significant differences in their internet behaviors.

Consistent with the model presented earlier, lonely people are predicted to spendmore time onS.N.S, prefer internet communications to face to face communications, and enjoy the anonymity and lurking more than others do. Increased pro-social behaviors in S.N.S result in their making friends, enjoying online friendships and feeling themselves more online. Lonely people are predicted to use S.N.S to modulate negative moods associated with loneliness.

Finally, lonely people are predicted to report disturbance in their life because of losing face to face interaction of real life;

H_1_-lonely and non-lonely individuals show significant difference in time spent in S.N.S.

H_2_-lonely and non-lonely individuals show significant difference in positive reasons for S.N.S use.

H_3_-lonely and non-lonely individuals show significant difference in negative reasons for internet use.

RQ_1_-Is there any significant difference in three aspects of Internet Behaviors (social aspect, negative impact & competency and convenience online)?

## 3. Method

### 3.1 Participants and Procedures

A survey that included time spent on S.N.S, “Internet Use Scale” and “Internet Behaviors Scale” (inspired by Morahan-Martin, 2003) as well as UCLA loneliness scale (Russel, 1996), was given to 156 Shiraz university students. Of these, 146 (93.6%) had experienced S.N.S use and were included in the research. 108 (69.2%) were female and 42 (26.9%) were male. The sample included 11 (7.1%) freshmen, 34 (21.8%) sophomore, 30 (19.2%) juniors, 6 (3.8%) seniors, 41 (26.3%) graduate student and 31 (19.9%) PHD students. The mean age of the participants were 24.59 (S.D=5.34). Participant reported an average weekly use as 18.01 hours (S.D= 20.38).

### 3.2 Measures

The questionnaires completed by the participants included demographic characteristics, S.N.S experience, reasons for S.N.S use, internet behaviors, and loneliness as follows:

#### 3.2.1 Demographic Characteristics

The demographic characteristics section of the questionnaire included questions on participants’ gender, and year in college.

#### 3.2.2 Internet Experience

The participants were asked if they had used S.N.S. Those who had used S.N.S were also asked about how many hours they used S.N.S per week.

#### 3.2.3 Reasons for Internet Use Scale

Positive and negative reasons for S.N.S use were assessed using “Reasons for internet use scale” (inspired by Morahan-Martin, 2003). This scale includes 17 Likert-type questions and four-point scale, with 1: Strongly disagree and 4: strongly agree. “The Reasons for Internet Use Scale” includes 7 positive reasons for S.N.S use and 10 negative ones. Reasons for S.N.S usewereassessed and showed a good reliability (coefficient α for positive reasons was as high as α = 0.786 and for negative reasons α =0.813with Iranian students). The result of exploratory factor analysis for this scale is presented in Table 1. As shown in Table 1, two dimensions (positive and negative reasons for S.N.S use) explained 42% of the total variation of the scale items. According to Table 2, all the 17 items of Reasons for S.N.S use Scale are located under relative dimension according to the factor analysis. The results indicate an appropriate validity with Iranian students. (Please see the appendix for more information on this measure).

**Table 1:** Results of exploratory factor analysis for reasons for internet use scale

Total Variance Explained									

Component	Initial Eigenvalues			Extraction Sums of Squared Loadings			Rotation Sums of Squared Loadings		
		
	Total	% of Variance	Cumulative %	Total	% of Variance	Cumulative %	Total	% of Variance	Cumulative %
1	4.972	29.249	29.249	4.972	29.249	29.249	4.383	25.782	25.782
2	2.219	13.054	42.304	2.219	13.054	42.304	2.809	16.521	42.304
3	1.584	9.320	51.623						
4	1.431	8.420	60.044						
5	1.270	7.472	67.516						
6	1.083	6.373	73.889						
7	.788	4.632	78.522						
8	.732	4.305	82.826						
9	.654	3.847	86.673						
10	.446	2.623	89.296						
11	.394	2.317	91.613						
12	.358	2.103	93.716						
13	.318	1.869	95.585						
14	.232	1.365	96.950						
15	.202	1.190	98.140						
16	.188	1.104	99.244						
17	.128	.756	100.000						

Extraction Method: Principal Component Analysis.

**Table 2:** Rotated component matrix

Component		

	1	2
**b1**	.344	.429
**b2**	**-.077**	**.785**
**b3**	-.419	.422
**b4**	**.622**	**-.172**
**b5**	-.640	.231
**b6**	**.541**	**-.034**
**b7**	-.416	.292
**b8**	**.114**	**.721**
**b9**	-.469	.535
**b10**	**.493**	**-.209**
**b11**	-.141	.691
**b12**	**.736**	**-.067**
**b13**	.712	.089
**b14**	**.571**	**.051**
**b15**	.511	-.037
**b16**	**-.734**	**.119**
**b17**	-.387	.551

#### 3.2.4 Scale of Internet Behaviors

Internet behaviors were assessed by 38 Likert-type questions that explore three aspects: social aspects of S.N.S use (19 questions), negative impact of S.N.S use (15 questions) and feeling of competency on line (4 questions inspired by Morahan-Martin, Schumacher, 2003). A four-point scale was used with 1 strongly disagree and 4: strongly agree. This scale assessed with a good reliability for three aspects as mentioned above (coefficient α_1_= 0.89 coefficient α_2_ =0.94 coefficient α_3_ =0.66) with Iranian university students. Convergent validity and discriminant validity of the scale of Internet Behavior was assessed (Table 3).

**Table 3:** Convergent validity and discriminant validity of the scale of internet behaviors

		Discriminant validity b		Convergent validity a	

Dimensions	Items	Range of correlation	Scaling success (percent)	Range of correlation	Scaling success (percent)
**Social aspect**	19	0.003-0.745	QUOTE 3838 38/38 (100)	0.011-0.791	17/19 (89.4)
**Negative impact**	15	0.011-0.139	QUOTE 2830 28/30 (93.3)	0.616-0.843	15/15 (100)
**Competency and convince**	4	0.019-0.819	7/8 (87.5)	0.472-0.845	4/4 (100)

a - Number of correlations between items and hypothesized scale corrected for overlap≥ 0.4,/total number of convergent validity test.

b - Number of convergent correlations significantly higher than discriminant correlations,/Total number of correlations.

These findings show that scaling success rates for convergent validity is 100% for all domains except for social aspects.The success rate for item discriminant validity of the Internet Behaviors Scale is 96.05 (73/76) (this statistical method for convergent and discriminant validity based on Fayers, 2000).

#### 3.2.5 UCLA Loneliness Scale

Loneliness was assessed using UCLA Loneliness Scale, version 3 (Rusell, 1996). This scale includes 20 Likert-type questions on a four-point scale, with 1- strongly disagree and 4= strongly agree. The UCLA loneliness scale has a good reported reliability coefficient α= 0.92 (Russell, 1996) and Validity. We also assessed reliability (coefficient α= 0.93) with university students in Shiraz. Bahirayi, Delavar and Ahadi (2007) reported a good validity and reliability for this scale with university students in Tehran. Loneliness was determined according to the total number of responses to these 20 questions.

The mean score was 40.39 (S.D=10.8). Students in the top 25% of the sample (n=41) scored 48 or higher were considered as lonely (L), and were compared with 25% of the sample of the sample with the lowest score (n=34) who were considered as non-lonely (NL). Their scores were less than 31. The mean score for lonelyand non-lonely individuals was 54.4 (SD=5.86) and26.2 (SD=2.67), respectively.

## 4. Results

### 4.1 S.N.S Use, Experience and Loneliness

No significant difference was found between lonely and non-lonely individuals in average weekly hours spent on S.N.S (t=0.35, P=0.972), with lonely users (M=18.91, S.D=21.86 and non-lonely users (M=19.13, SD=26.82). Therefore, H1wasnot confirmed. The resultsare presented in Table 4.

**Table 4:** Difference between lonely and lonely individuals in hours spent on S.N.S

	n	Mean	SD	t	df	P
**L**	34	18.91	21.86	0.035	61	0.972
**NL**	29	19.13	26.82			

### 4.2 Reasons for S.N.S. Use and Loneliness

To explore differences between lonely and non-lonely S.N.S use independent sample t-test was conducted. There was no significant difference between lonely and non-lonely individuals for positive and negative reasons for S.N.S use. Therefore, H2 and H3 were also not confirmed. The results are presented in Tables 5 and 6.

**Table 5:** Difference between lonely and non-lonely individuals in negative reasons for S.N.S use

	n	Mean	SD	t	df	P
**L**	41	29.44	9	-0.501	73	0.618
**NL**	34	28.29	10.8			

p<0.05*; p<0.01**

**Table 6:** Difference between lonely and non-lonely individuals in positive for S.N.S use

	n	Mean	SD	t	df	P
**L**	41	17.97	5.02	-1.39	73	0.167
**NL**	34	16.18	6.15			

p<0.05*; p<0.01**

### 4.3 Internet Behaviors and Loneliness

To explore differences, independent samples t-test was conducted between lonely and non-lonely individuals in “social aspect”, “negative impact” and competency and convenience of internet behaviors (Please see the appendix for more information on these dimensions). There was a significant difference between lonely and non-lonely in social aspect and negative impact of internet behaviors but no difference was seen in competency and convenience aspect of internet behaviors. The results are present in Tables 7, and 8.

**Table 7:** Difference between lonely and non-lonely individuals in social aspect of internet behaviors

	n	Mean	SD	t	df	P
**L**	34	31.4	10.07	-40.7	73	**0.000
**NL**	41	40.92	10.08			

p<0.05*; p<0.01**

**Table 8:** Difference between lonely and non-lonely individuals in negative impact of internet behaviors

	n	Mean	SD	T	df	P
**L**	34	22.74	6.68	-5.48	73	**0.000
**NL**	41	34	10.3			

p<0.05*; p<0.01**.

**Table 9:** Difference between lonely and non-lonely individuals in competency and convenience of internet behaviors

	n	Mean	SD	t	df	P
**L**	34	11.35	2.74	0.347	72	0.729
**NL**	41	11.17	1.6			

p<0.05*, p<0.01**

Hence, asmentioned earlier, social aspect and negative impact showed a significant difference between lonely and non-lonelyindividuals.

## 5. Discussion

The present study did not confirm the hypothesis that lonely people (in comparison to non-lonely people) spend significantly more time on S.N.S. Also, their positive and negative reasons for S.N.S use showed no significant difference (contrary to literature). This similarity between lonely and non-lonely individuals in recent years could be justified by the great changes in social norms. It seems like people do notattend S.N.S because theyfeel lonely. For most people S.N.S is a new way to constructsocial Identity. They can also present themselves much closer to the “Ideal self” (that is why people share their best pictures and moments to get more likes), and they end up with distortions of what they really are. Furthermore, S.N.S provides them with advanced and easy access interactions, which is attractive for everyone because it is cheap and fast.

Although there was no difference in reasons for S.N.S use between lonely and non-lonely individuals, after attending S.N.S, they show significantly different behaviors.

Social behavior of lonely individuals showed significant difference in comparison to non-lonely. It was consistently enhanced online, along with the disinhibition effect. Lonely individuals were more likely to prefer communication, to lurk and to enjoy the anonymity of online communications. They felt more themselves, opened up more and were friendlier compare with non-lonely individuals. Lonely individuals felt that going online had made it easier for them to make friends. These are examples of behaviors that make lonely people significantly different from non-lonely individualsinsocial aspects of internet behaviors. Disinhibition, anonymity and capacity to select target audience explain why the present study in line with other studies has found that some users feel more themselves online than offline. Mckenna (1989) refers to this as the “real me online” and has documented that this phenomena is associated with loneliness.

This study also confirmed that lonely people significantly feel negative impacts of using S.N.S which is in agreement with other studies (Lavin et al., 1999; Loytskert & Aiello, 1997; Morahan-Martin & Schumacher, 2003; Ghasemi et al., 2007). Ironically, although lonely users enjoy enhanced social behavior online, their use of S.N.S interferes with *offline activity*, as well as occupational adjustment and caused guilt as reported by the study participants. Lonely individuals were more likely to use social aspect of S.N.S when they feel lonely, down, or anxious, so there is a vicious circle whereby lonely individuals go online to fill social voids in their life, but time spent on S.N.S creates voids in their offline lives, and creates other life problems.

## 6. Conclusion

This study has suggested that there is no significant difference between lonely and non-lonely individuals in reasons for S.N.S use and time spent onS.N.S (contrary to the literature).In other words, what drives people to S.N.S at the first place shows no significant difference between lonely and non-lonely individuals any more. But after attending S.N.S, social behaviors of lonely individuals show significant differences and were *consistently enhanced online*. Lonely people also significantly develop internet-related problems in their daily functioning, including interference with real life socializing. Nevertheless, some studies suggest that internet can provide a safe area for individuals to develop and practice social skills (Kandell, 1998; Suler, 1996; Turkle, 1984). It seems that further research isneeded on the effects of S.N.S on daily life and mental health especially in the Iranian society due to a few numbers of studies.

This study is preliminary and raises many avenues for future research on changing patterns of online behavior for both lonely and non-lonely individuals.Do lonely people who use S.N.S show the same pattern as Obsessive Compulsive Disorder (OCD)? Does their S.N.S usework like neutralizing behaviors which help them to experience less anxious primarily, but end them up with more anxiety and disturbance interpersonally? The questions of cause and effect in terms of loneliness and S.N.S use, as well as whether state against trait loneliness interact with S.N.S use still require more studies. Longitudinal research on more diverse population is suggested.

## Appendix:

**Appendix A**

**Reasons (positive and negative) for internet use scale**

1- I use S.N.S to communicate with friends and family. (+)
2- I use S.N.S for required course work. (+)
3- I use S.N.S for recreation. (+)
4- I feel relaxed just by using S.N.S. (-)
5- I use S.N.S to find job most of time. (-)
6- I make new friendship just by using S.N.S (-)
7- I talk to others who share common interests only through S.N.S. (-)
8- I use S.N.S for staying abreast of new development in areas of interests. (+)
9- I use S.N.S for sharing ideas or fantasies. (+)
10- I use S.N.S for wasting time. (-)
11- I use S.N.S for finding information for own use. (+)
12- I use S.N.S seeking emotional supports. (-)
13- I use S.N.S for gambling (-)
14- I use S.N.S for net resources intended for adults only. (-)
15- I use S.N.S for games. (-)
16- I just use S.N.S for virtual Reality. (-)
17- I use S.N.S for browsing. (+)
+ marks positive reasons
- marks negative reasons

**Appendix B**

**Scale of Internet Behaviors (Social Aspect)**

1- My online friends understand me better than other people.
2- I am more myself online that in real life.
3- I open up more to people online than in other communications modes.
4- Most of my friends I know from online.
5- I prefer communication online to face to face communication.
6- I am friendlier online than in real life.
7- The anonymity of being online is liberating.
8- I have shared intimate secrets online.
9- I have lurked online but never entered a conversation online.
10- Going online has made it easier for me to make friends.
11- I have more fun with the people I know online than others.
12- I have a network of friends made online.
13- Sometime I pretend I am someone I am not while online.
14- I like the speed of communication online.
15- I prefer telephoning to communicating online. (reverse)
16- Online communication lets me control when I want to communicate.
17- I have pretended to be somebody of the opposite sex while online.
18- Being online has made it easier to communicate with people I know.
19- I feel less connected interpersonally when I communicate online. (reverse)

**Appendix C**

**Scale of Internet Behaviors (Negative Impact)**

1- I feel guilty about time spent online instead of at other require work.
2- I have been told that I spend too much time online.
3- I have routinely cut short on sleep to spend more time online.
4- I have gone online to make myself feel better when down or anxious.
5- I have use online to talk to others when I was feeling isolated.
6- I have missed social engagements because of online activities.
7- I have missed classes or work because of online activity.
8- I have attempted to spend less time online but have not being able to.
9- When I am online, I feel totally absorbed.
10- If it has been along times since I last logged on, I find it hard to stop thinking about what will be waiting for me when I do.
11- I have tried to hide from others how much time I am actually online.
12- I have gotten in to trouble with my employer or school because of being online.
13- I sometime go online to escape from pressure.
14- I have never gotten in to an argument with a significant other over being online.
15- My work and/or school performance has not deteriorated since I started going online.

**Appendix D**

**Scale of Internet Behaviors (Competency and Convenience Aspect)**

1- I avoid going online for information because there is too much to weed through. (reverse)
2- I feel competent in my ability to use online services.
3- I am comfortable using online services.
4- Going online has made it easier for me to do research.
